# 4D Electromagnetic Navigation Bronchoscopy for the Sampling of Pulmonary Lesions: First European Real-Life Experience

**DOI:** 10.1007/s00408-021-00477-z

**Published:** 2021-09-25

**Authors:** Filippo Patrucco, Matteo Daverio, Chiara Airoldi, Zeno Falaschi, Vittorio Longo, Francesco Gavelli, Renzo Luciano Boldorini, Piero Emilio Balbo

**Affiliations:** 1 Respiratory Diseases Unit, Medical Department, AOU Maggiore Della Carità, C.so Mazzini 18, 28100 Novara, Italy; 2 Department of Translational Medicine, University of Piemonte Orientale, Novara, Italy; 3 Radiodiagnostics, Department of Diagnosis and Treatment Services, AOU Maggiore Della Carità, Novara, Italy; 4 Pathology Unit, University of Piemonte Orientale, AOU Maggiore Della Carità, Novara, Italy

**Keywords:** Electromagnetic Navigation Bronchoscopy, Pulmonary Nodule, Pulmonary Masses, Navigation System, Bronchus Sign, Lung Cancer Diagnosis

## Abstract

**Purpose:**

The use of Electromagnetic navigation bronchoscopy (ENB) for the diagnosis of pulmonary peripheral lesions is still debated due to its variable diagnostic yield; a new 4D ENB system, acquiring inspiratory and expiratory computed tomography (CT) scans, overcomes respiratory motion and uses tracked sampling instruments, reaching higher diagnostic yields. We aimed at evaluating diagnostic yield and accuracy of a 4D ENB system in sampling pulmonary lesions and at describing their influencing factors.

**Methods:**

We conducted a three-year retrospective observational study including all patients with pulmonary lesions who underwent 4D ENB with diagnostic purposes; all the factors potentially influencing diagnosis were recorded.

**Results:**

103 ENB procedures were included; diagnostic yield and accuracy were, respectively, 55.3% and 66.3%. We reported a navigation success rate of 80.6% and a diagnosis with ENB was achieved in 68.3% of cases; sensitivity for malignancy was 61.8%. The majority of lesions had a bronchus sign on CT, but only the size of lesions influenced ENB diagnosis (*p* < 0.05). Transbronchial needle aspiration biopsy was the most used tool (93.2% of times) with the higher diagnostic rate (70.2%). We reported only one case of pneumothorax.

**Conclusion:**

The diagnostic performance of a 4D ENB system is lower than other previous navigation systems used in research settings. Several factors still influence the reachability of the lesion and therefore diagnostic yield. Patient selection, as well as the multimodality approach of the lesion, is strongly recommended to obtain higher diagnostic yield and accuracy, with a low rate of complications.

## Introduction

The early detection and diagnosis of pulmonary lesions represents the cornerstone in lung cancer mortality reduction [1]. Electromagnetic navigation bronchoscopy (ENB) provides a multiplanar approach to lung lesions, leading the bronchoscope in close proximity for sampling procedures [2]. The navigation bronchoscopy system allows the bronchoscopist to better find the correct route to the target pulmonary lesion, compared to the conventional fluoroscopy-guided bronchoscopy [3]. Over the last years, many studies have been published on this subject, showing a pooled diagnostic yield of ENB between 65% and 74%, with a sensitivity of 77% [4–6]. The variability of the ENB diagnostic yield is influenced by many factors: some are dependent on the characteristics of lesions such as size, lobar location, presence of bronchus sign [3, 7–13], whereas some depend on both navigation system and tools used, or are operator dependent [14–17]. The results of a large real-life study reported a low diagnostic yield for endobronchial navigation system, even when its use was associated with radial endobronchial ultrasound (r-EBUS), questioning about the real performances of navigation system [3]. Nevertheless, a recent meta-analysis confirmed a higher diagnostic yield of navigation bronchoscopy systems for pulmonary nodules, 1.69 times higher than other non-navigation bronchoscopy ones [18, 19].

Recently, a new ENB system based on 4D technology was introduced to approach peripheral lesions: the 4D ENB was developed to overcome the respiratory motion, reducing the inaccuracies of previous ENB systems in sampling procedures of nodules moving during respiratory cycle [20]. A pre-procedure CT collects images during the inspiratory and expiratory phases and then, during the airway inspection, the sensor probe collects 3D points reconstructing both the lumen registration map and the pathway to the target lesion. Pulmonary nodules location was demonstrated to be closer on expiratory phase acquisition images than inspiratory ones, suggesting a coordination during expiratory phases of sampling procedure [21]. Moreover, this technology incorporates the electromagnetic guidance system to perform a transthoracic needle aspiration (TTNA) sampling by using the same CT images. This approach had a diagnostic yield of 83%, up to 87% when TTNA was combined with the same-procedure ENB [22].

The primary aim of our study is to report the 4D SPiN® Thoracic Navigation System feasibility, diagnostic yield, accuracy and safety in approaching pulmonary lesions; the second aim is to evaluate factors that could influence the diagnostic performance of this navigation system.

## Materials and Methods

This study was conducted in accordance with the STROBE statement for observational studies [23]. Our local institutional Review Board approved of the study. All the procedures performed in this study were in accordance with the 1964 Helsinki declaration and its later amendments or comparable ethical standards.

### Patients

We conducted a single-centre retrospective observational study, including all patients with pulmonary nodules or masses who underwent ENB between 24th July 2018 and 30th September 2020 at the Interventional Pulmonology Unit of Maggiore della Carità Hospital in Novara, Italy.

Demographic data, main findings of pulmonary lesions at CT scan images were recorded for each patient. In particular, for each lesion we specified size, localization, distance to the pleura (visceral pleura), different type of bronchus sign (type A when the responsible bronchus clearly reaches the inside of the target lesion, type C when no bronchus can be detected in relation to the lesion, type B when the CT finding cannot be categorized either into type A or type C [24]), lesions’ standardized uptake values (SUV) at positive emission tomography/CT (PET/CT). For each lesion, we also evaluated whether it was previously sampled during a conventional fluoroscopy-guided bronchoscopy or directly approached with ENB. For each lesion sampled during ENB, we recorded which sampling tool was used (transbronchial needle aspiration, TBNA, transbronchial lung biopsies with forceps, TBLB, bronchoalveolar lavage, BAL), the diagnosis achieved with the ENB procedure and, in case of a negative ENB, which other procedure reached a final diagnosis.

### Diagnostic Pathway

As previously published, ENB was used as a second step of diagnostic approach when the patients previously underwent a non-diagnostic fluoroscopy-guided bronchoscopy [2]. We reserved ENB as first approach in selected difficult cases (e.g. presence of bronchus sign, lesions located in the upper lobes, diameter smaller than 20 mm), with a high risk of procedure-related complications (i.e. lesions surrounded by emphysema, chronic respiratory failure), with undetectable lesion by conventional fluoroscopy or, finally, according to the preference of the patient [2].

In the case of non-diagnostic ENB, the patients underwent either an additional diagnostic procedure (i.e. fluoroscopic or CT-guided TTNA or surgical biopsy) or a clinical and radiological follow-up, until a final diagnosis was achieved.

### Procedures

**Fig. 1:**
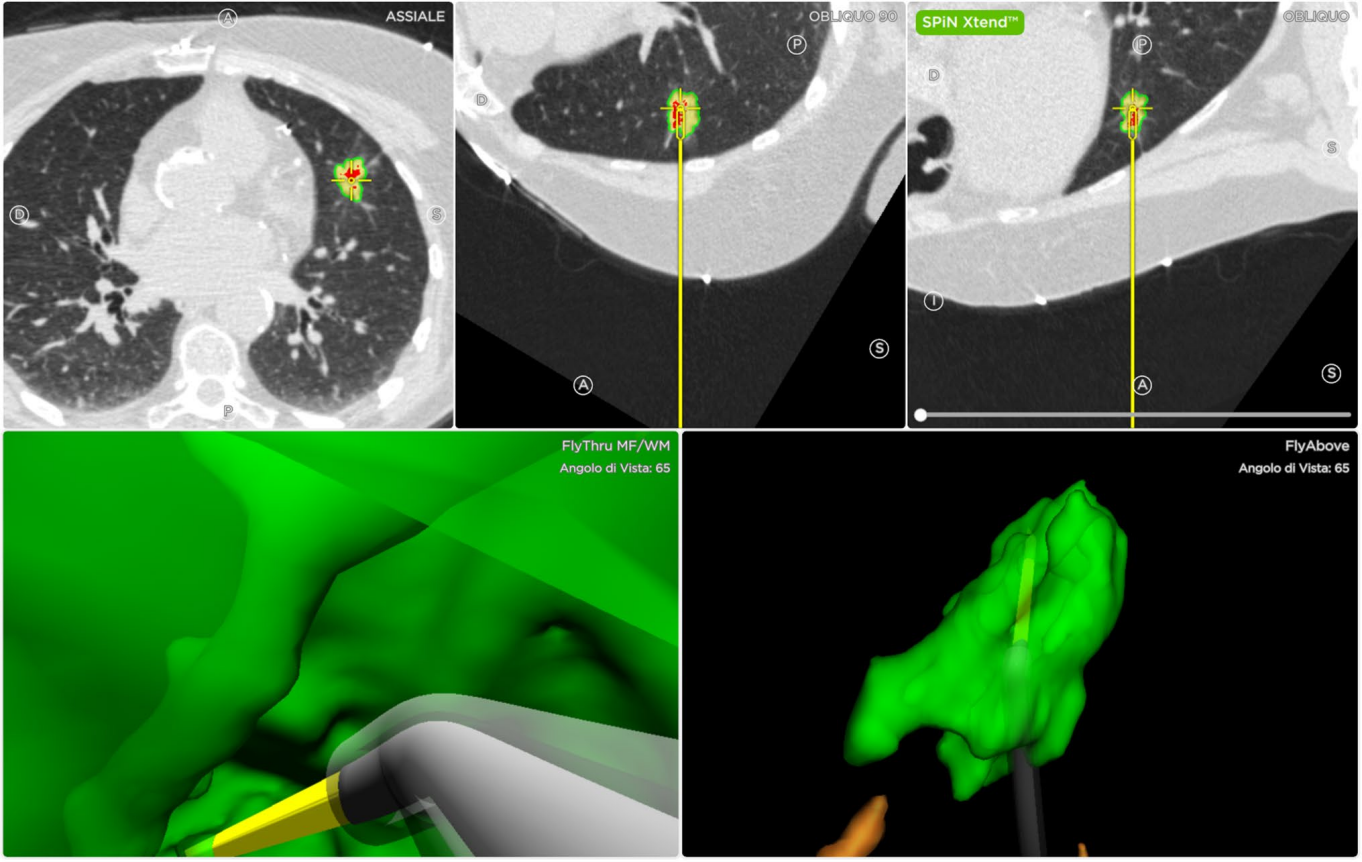
Multiplanar Computed Tomography images (upper images), 3D images of the real-time sampling with transbronchial needle aspiration (lower images)

The day of the procedure patients underwent a chest CT scan (0.5 mm interval, 0.75 mm thickness), with acquisition images at maximal inspiratory breath hold and expiratory breath hold at functional residual capacity, as previously reported [22]. The post processing of the acquired images generated a virtual airway map (Veran Medical Technologies, Inc., St. Luis, MO, USA) after the placement of a navigational tracking pad (vPAD2, Veran Medical Technologies, Inc.) on patients’ anterior chest. The bronchoscopist, then, identified the target lesion and an endoscopic planning route was generated by the software (Fig. 1).

All the procedures were performed under general anaesthesia by a single operator (PEB), highly experienced in ENB procedures (the operator performed more than 200 ENB procedures with different ENB systems), using the SPiN® Thoracic Navigation System (Veran Medical Technologies, Inc.). No other guidance systems were used for all sampling procedures (i.e. fluoroscopy or r-EBUS). Target lesions were reached using an electromagnetic tip-tracked biopsy instrument (21 Gauge Needle and 1.8-mm outer diameter Serrated Cup Always-On Tip Forceps, Veran Medical Technologies, Inc.) inserted in the working channel of a therapeutic bronchoscope (Olympus BF-H190, except for three cases where Olympus BF-MP190F was used). Once reached, the lesion was sampled with a hierarchical approach: a maximum of 4 passages of TBNA were followed by up to 4 TBLB with forceps and, finally, a selective BAL with a 50-mL sterile saline was performed. In our cohort, after each TBNA sampling, we always performed a rapid on site evaluation (ROSE), in order to decide whether or not to proceed with TBLB, providing more tissue for the pathology evaluations [2].

For each ENB procedure, we reported the sampling tools used and potential complications (pneumothorax, haemoptysis, respiratory distress).

### Statistical Analysis

Categorical variables are presented as absolute value and percentage, while for continuous ones we reported mean ± SD or median and interquartile range [IQR], as appropriate. Statistical comparisons between ENB diagnosis and categorical variables were made using chi-square test, while t-student or non-parametrical tests were used for the continuous ones. Navigation success (number of lesions reached by ENB over all target lesions), diagnostic yield (malignancies and benign diagnoses over all target lesions), diagnosis accuracy (malignancies, benign diagnoses and intermediate results confirmed correct over all sampled nodules with known final diagnosis) and sensitivity for malignancies (malignancies over the final number of malignancies after further testing) were then calculated [4]. Navigation success was defined when the diagnostic marked tool (needle or forceps) reached the surface of the lesion, highlighting the target lesion with green colour. The 95% confidence interval [95% CI] was also reported. A *p* value < 0.05 was considered as statistically significant. Statistical analysis was performed using SAS 9.4. (SAS Institute Inc., Cary, NC, US).

## Results

One-hundred-three ENB sampling procedures were performed among 77 subjects. Most patients were male (68.8%) with a median age of 72.6 years (minimum 39.13, maximum 86.99 years). The lesions were mainly located in the upper or middle lobes (79.6%) and were solid (79.6%) with spiculated margins (52.4%). Bronchus sign pattern was type A in 28.1%, B in 52.4% and C in 19.4% of cases. Most sampled lesions were nodules (61.1%), with a median maximum diameter of 26 mm, and were located in the outer diameter of the lung parenchyma (median distance to the visceral pleura of 4 mm). Median PET/CT SUV was 5.96 [3.16–9.33] g/mL; 45 patients (58%) had a prior negative fluoroscopy-guided bronchoscopy (Table 1).

**Table 1:** Demographics and lesions’ characteristics

	*N* (%)
Patients (*n* = 77)	
Gender	
Male	53 (68.83)
Female	24 (31.17)
Age, years	
Mean ± SD	72.59 ± 8.28
Lesions (n = 103)	
Location	
RLL e LLL	21 (20.39)
RUL, LUL, ML	82 (79.61)
Type of lesions	
Solid	82 (79.61)
Ground glass opacity	1 (0.97)
Part-solid part-ground glass	20 (19.42)
Spiculated	
Yes	54(52.43)
Bronchus sign type	
A	29 (28.16)
B	54 (52.43)
C	20 (19.42)
Lesions with maximum diameter, mm	
< 20	26 (25.24)
20–30	37 (35.92)
> 30	40 (38.83)
Mean ± SD	28.91 ± 14.30
Median [IQR]	26 [20–38]
PET, SUV	
Mean ± SD	6.54 ± 3.65
Median [IQR]	5.96 [3.16–9.33]
Pleura distance, mm	
Mean ± SD	8.03 ± 10.47
Median [IQR]	4.00 [0–14]

*LLL* Left Lower Lobe, *LUL* Left Upper Lobe, *ML* Middle Lobe, *PET* Positron Emission Tomography, *RLL* Right Lower Lobe, *RUL* Right Upper Lobe, *SUV* Standardized Uptake Value

ENB allowed to reach the lesion in 83 cases (navigation success 80.6%) and the diagnosis was achieved with ENB in 57 cases (57/83 = 68.3%). The final diagnosis was definitively achieved with other techniques in 16 cases: 13 with surgery and 3 with TTNA. The diagnosis was achieved in 70.9% of the sampled lesions (73/103). For the remaining 30 lesions, 11 were lost at follow-up, 13 were considered malignant because the subsequent CT control demonstrated an enlargement of lesions’ diameter, 1 lesion was stable after 2 years of CT follow-up, whereas 5 patients are still in follow-up.

The diagnostic yield and accuracy of ENB were, respectively, 55.3% (57/103) and 66.3% (57/86); sensitivity for malignancy was 61.8% (47 malignancies diagnosed with ENB/76 total malignances).

Malignancies were 40 lung adenocarcinomas, 7 lung squamocellular, 7 neuroendocrine lung tumours, 2 large cells lung carcinoma, 1 mesenchymal cell tumour, 1 primitive pulmonary lymphoma, 5 metastases and 13 non-defined histotype. Definitive diagnosis of benign lesions were 4 inflammatory and 4 fibrotic infiltrates (confirmed at further CT or PET), 1 eosinophilic pneumonia and 1 aspergillosis; 1 lesion was stable after 2 years of follow-up.

Among the characteristics of lesions, only size influenced the diagnostic performance of ENB, with diagnosed lesions having a median maximum diameter of 28 mm (*p* = 0.0201). Other characteristics were not associated with ENB diagnosis (Table 2); even if non statistically significant, nearly 80% of ENB diagnostic procedures were performed on lesions located in the upper-middle lobes, with a solid pattern and had a diameter greater than 20 mm.

**Table 2:** Factors influencing ENB diagnosis

	ENB non-diagnostic (*n* = 46)	ENB diagnostic (*n* = 57)	*p* value
Location			
RLL e LLL	9 (19.57)	12 (21.05)	0.8522
RUL, LUL, ML	37 (80.43)	45 (78.95)	
Type of lesions			
Solid	38 (82.61)	44 (77.19)	0.3188
Ground glass opacity	1 (2.17)	0	
Part-solid part-ground glass	7 (15.22)	13 (22.81)	
Spiculated			
Yes	20 (43.48)	34 (59.65)	0.1023
Bronchus sign type			
A	8 (17.39)	12 (21.05)	0.8478
B	14 (30.43)	15 (26.32)	
C	24 (52.17)	30 (52.63)	
Lesions with maximum diameter, mm			
< 20	14 (30.43)	12 (21.05)	0.2676
20–30	18 (39.13)	19 (33.33)	
> 30	14 (30.43)	26 (45.61)	
Median [IQR]	23 [20–32]	28 [21–40]	0.0201
Pleura distance, mm			
Median [IQR]	7.50 [0–20]	0 [0–10]	0.1117
Definitive diagnosis (N = 86)			
Malign	29 (100.00)	47 (82.46)	0.0143
Benign	0	10 (17.54)	

*ENB* Electromagnetic Navigation Bronchoscopy, *LLL* Left Lower Lobe, *LUL* Left Upper Lobe, *ML* Middle Lobe, *RLL* Right Lower Lobe, *RUL* Right Upper Lobe

The majority of the lesions were sampled with all instruments (63%, TBNA, TBLB and BAL): TBNA was used 93.2% of times, whereas TBLB and BAL 79.6% and 81.6% of times, respectively. TBNA was the tool that mainly contributed to ENB diagnosis, accounting for 70.2% (40/57), TBLB for 56.1% (32/57) and BAL only 24.6% (14/57) (Table 3).

**Table 3:** Sampling tools used

Procedure	% use (*n* = 103)	*N* positive/total lesions	*N* positive/ENB positive	% IC 95%
TBNA	96 (93.20%)	40/103 (38.83%)	40/57 (70.17%)	41.67 [31.80–51.53]
TBLB	82 (79.61%)	32/103 (31.06%)	32/57 (56.14%)	39.02 [28.47–49.58]
BL	84 (81.55%)	14/103 (13.59%)	14/57 (24.56%)	16.67 [8.70–24.64]
TBNA or TBLB or BL		56/103 (54.36%)		54.37 [44.75–63.99]

*BL* Bronchial Lavage, *ENB* Electromagnetic Navigation Bronchoscopy, *TBLB* Transbronchial Lung Biopsy, *TBNA* Transbronchial Needle Aspiration

We observed only one case of pneumothorax, which did not require to be drained. No other procedure-related complications were recorded.

## Discussion

In our cohort, we report diagnostic performances of a 4D navigation system platform for diagnosis of peripheral pulmonary lesions. The diagnostic yield and accuracy were, respectively, 55.3% and 66.3% with a sensitivity for malignancy of 61.8%. These results, acquired without other guidance tools, are slightly lower than the previously reported ones for other navigation systems used in research settings [4]. Nevertheless, they are in line with, or even higher than, real-life studies, where diagnostic yield reaches 38%, questioning the role of navigation systems for the diagnosis of pulmonary nodules in real-life settings [3]. Raval et al. used the SPiNDrive system on 49 patients with 61 lesions with a majority of pulmonary nodules; they reported an overall diagnostic yield of 83.3% [19]. The results of a recent meta-analysis, evaluating the value of navigation bronchoscopy for the diagnosis of peripheral pulmonary lesions, underlined that the diagnostic yield of navigation bronchoscopy was higher than non-navigation bronchoscopy approaches, with an overall odds ratio of 1.69 [18].

There are many factors that could influence ENB diagnostic yield: lesion size, lobe location, distance from the pleural surface, presence of a bronchus sign and malignant nature of the lesion are those that mainly influence diagnostic yield [18]. In particular, pulmonary lesions with diameter < 20 mm were more frequently diagnosed with navigation bronchoscopy systems than non-bronchoscopy ones (64.09% versus 48.67%) [18]. Other previously published studies identified both the size and the bronchus sign as factors influencing ENB performance [13]. In the study of Raval et al. [19], bronchus sign was present in 52% of cases and, among them, diagnosis was achieved 88% of times. By contrast, even in the absence of a bronchus sign, a diagnosis was achieved in 78% of cases [19]. In our study, we found that the only characteristic associated with better ENB performance was size, with a median diameter of 28 mm in diagnosed lesions. We did not confirm our previously published results, where a bronchus sign was the only factor associated with a higher diagnostic yield [2]. This may be influenced by a bias in patient selection: in order to maximize the pre-test probability to have a diagnostic ENB by having many patients with lesions in the upper lobes and with a bronchus sign, in our cohort most of the sampled lesions had a bronchus sign (80.58%) and were located in the upper or middle lobes (79.6%).

The 4D SPiN® Thoracic ENB System allows the physician to overcome some crucial limits of previous ENB systems. Firstly, the motion of pulmonary lesions during the respiratory cycle, especially when they are located in the lower lobes: the acquisition of inspiratory and expiratory CT sequences allows a better virtual reconstruction of the endobronchial pathway to the lesion. Secondly, the acquisition of CT on the same day of the bronchoscopic procedure could reveal last-minute variations in the characteristics of lesions [21].

The NAVIGATE post hoc analysis confirmed that in the multimodal approach strategy to the nodule, the aspirating needle and forceps had higher true-positive rates [6, 25]. The Acquire registry reported that TBNA improved diagnostic yield when compared with other diagnostic tools, such as forceps biopsy, transbronchial brushing and lavage [3]. In our cohort, the extensive approach with all three instruments (TBNA, TBLB and BAL) was used 63% of times and TBNA was the mostly used tool (93% of times) with the higher diagnostic yield (70%); other sampling tools had lower diagnostic rates, even if they were used, beside TBNA, nearly 80% of times.

Another factor that could influence positively the diagnostic rate is the use of an ultra-thin bronchoscope. Ali et al. achieved a 90% diagnostic yield using an ultra-thin bronchoscope in combination with Cone Beam CT [24]. In our cohort, we used an ultra-thin bronchoscope only three times and the navigation success was 100%: the tip of the bronchoscope could always reach the lesion under ENB guidance and all three lesions were sampled. The main difference is the manoeuverability of an instrument with a diameter of 3.0 mm in a combined working channel of 1.7 mm (Olympus BF-MP190F) against standard bronchoscope (Olympus BF-H190) with a diameter of 5.0 mm and a working channel of 2.0 mm.

The transthoracic approach to pulmonary lesions is generally performed under CT guidance processing multiple samplings to achieve a real-time diagnosis. Even though the diagnostic yield is higher than bronchoscopy without guidance systems, the number of procedure-related complications is higher, with 16% of pneumothorax and 1% of major haemorrhage [26]. The introduction of the SPiN System™ allows the pulmonologist to biopsy a pulmonary lesion by performing a single percutaneous passage under electromagnetic guidance alone [22]. Mellow et al. in a retrospective analysis of 129 procedures using SPiNPerc™ for transthoracic sampling of pulmonary nodules reported a diagnostic yield of 73%, which raised to 81% when it was combined with ENB [27]. The reported complication rate in their study was 22.5%, 17% of which were pneumothorax [27]. In our cohort, we never used this approach: the implementation of transthoracic sampling, possibly during the same procedure, taking advantage of ROSE, would ideally achieve a diagnosis even in those three patients who subsequently underwent a diagnostic TTNA.

We also confirmed the low incidence of procedure-related complications. We reported only one case of pneumothorax that resolved spontaneously. In literature, the prevalence of complications was 3.2% and the most common complications were pneumothorax (1.7%) and haemorrhage (1.38%) [18].

Major limitations of our study are as follows: the retrospective nature and consequently the absence of a control group for comparison analysis; we did not have an r-EBUS in use as additional guidance tool, circumstance which limited the possibility to better define interrelations between bronchus lumen and lesions, in particular, for those lesions with a type B bronchus sign. The inflexibility of forceps and needles as well as the use of operative bronchoscopes (large diameters of bronchoscope’s tip, anatomical bronchial angulations) may have influenced our diagnostic performances. The rate of navigation success: our results are lower than those reported in literature [28]; such results are probably conditioned by technical and anatomical aspects (i.e. number or bronchial division, strict bronchial angulations); the implementation with other guidance tools (i.e. r-EBUS or guide sheath, unavailable at our institution) could improve the navigation success rate. Moreover, among those procedures that we defined as non-success four were definitively diagnosed as benign lesions; considering these lesions as diagnosed with ENB the diagnostic yield would slightly increase. Moreover, we defined navigation success when the tip of the sampling tool reached the lesion’s surface; we based this definition on a virtual image, not in real life. The combination of other guidance tools (i.e. r-EBUS, cone beam CT) would probably increase the rate of navigation success and consequently the diagnostic yield. The acquisition of the CT on the same day of the bronchoscopic procedure needs a great coordination between all the involved services (i.e. radiologists, anaesthesiologists, pulmonologists). Finally, the diagnostic pathway designed in our institution could have influenced the selection of patients, as well as the pre-test probability of lung cancer. However, to the best of our knowledge, this is the first European report of a large real-life cohort of patients undergoing bronchoscopy with the use of 4D SPiN® Thoracic Navigation System for sampling pulmonary nodules and masses.

## Conclusions

In conclusion, with our real-life study, we reported a diagnostic yield of 55% and an ENB diagnostic rate of 68% for the sampling of pulmonary lesions and masses; these results are lower than those previously reported in the literature using other guidance tools. The selection of patients and lesions (upper-middle lobes, diameter greater than 20 mm, solid), as well as the use of all the sampling tools in combination, provide better results in absence of the risk of major complications. CT acquired the same day of the procedure, with acquisition of inspiratory and expiratory scans, could help bronchoscopist during the sampling with a better coordination, in phase with respiratory motion, although this not fully overcomes all the challenges in peripheral sampling.
